# An update on migraine: current understanding and future directions

**DOI:** 10.1007/s00415-017-8434-y

**Published:** 2017-03-20

**Authors:** Francesca Puledda, Roberta Messina, Peter J. Goadsby

**Affiliations:** 1 Headache Group, Department of Basic and Clinical Neuroscience, King’s College London, London, UK; 2 NIHR-Wellcome Trust King’s Clinical Research Facility, King’s College Hospital, London, UK

**Keywords:** Migraine, Pathophysiology, Treatment, CGRP, Neuromodulation

## Abstract

Migraine is a common brain disorder with high disability rates which involves a series of abnormal neuronal networks, interacting at different levels of the central and peripheral nervous system. An increase in the interest around migraine pathophysiology has allowed researchers to unravel certain neurophysiological mechanisms and neurotransmitter involvement culminating in the recent development of novel therapies, which might substantially change the clinical approach to migraine patients. The present review will highlight the current aspects of migraine pathophysiology, covering an understanding of the complex workings of the migraine state and the brain regions responsible for them. We will further discuss the therapeutic agents which have appeared in the most recent years for migraine care, from calcitonin gene-related peptide (CGRP) receptor antagonists, *gepants*; through serotonin 5-HT_1F_ receptor agonists, *ditans*, and CGRP or CGRP receptor monoclonal antibodies to invasive and non-invasive neuromodulation techniques.

## Introduction

Migraine is the most common neurological cause of disability in the world [1]. Notwithstanding, clinicians and researchers have seen little progress in the therapeutic options available to treat this condition in the last two decades. Recent advances in our understanding of migraine pathophysiology have allowed the development of pharmacological and non-pharmacological treatments that offer the advantage of targeting mechanisms known to be active in the disorder leading to better management of patients.

The current review follows this bench to bedside approach [2], with an outline of relevant mechanisms in migraine biology, followed by an up-to-date summary of the most important therapies used in migraine at the present stage.

## Migraine pathophysiology

Over the last two decades our knowledge of the biology of migraine has improved considerably, with a series of basic science and imaging studies that demonstrate how vascular changes, first thought to explain migrainous pain, are neither necessary, nor sufficient in migraine [3, 4]. From a vascular theory the field has moved on to Neuronal theories involving the central or peripheral nervous system, or both. Much research has focused on specific brain structures thought to be at the basis of pain, arguably the primary migraine symptom. With these advances, it has become clear that the concept of a unique migraine generator may not be useful, in view of the variety of overlapping phases that constitute the migraine attack.

It is now widely accepted that migraine should be viewed as a complex brain network disorder with a strong genetic basis that involves multiple cortical, subcortical and brainstem regions to account for the pain and the wide constellation of symptoms characterizing the attack [4–6]. Here we will describe some important advances in our understanding of the different brain areas known to be directly involved in the premonitory, aura, pain and postdromal phases of migraine.

### The trigeminal vascular system and brainstem nuclei

The trigeminovascular system is one of the key actors in the expression of migraine headache. It consists of peripheral axons from the trigeminal ganglion that reach the meninges and intracranial arteries and converge centrally in the trigeminocervical complex releasing, among other transmitters, calcitonin gene-related peptide (CGRP) [7, 8]. The trigeminocervical complex (TCC) consists of the trigeminal nucleus caudalis along with the dorsal horn of C1–C2 segments of the spinal cord [9, 10]. Its activation is thought to lead to the cascade of events resulting in the migraine pain due to its direct connection with key brain centres such as diencephalic and brainstem nuclei [11, 12].

In the late 1980s it was proposed that migraine pain may be due to a sterile neurogenically induced inflammation of the dura mater [13, 14]. However, the failure of specific plasma protein extravasation blockers as acute or preventive migraine treatments in randomized controlled trials suggested other explanations were needed [15, 16]. Human observational [17] and brain imaging studies [18–20] have suggested a role of brainstem regions, such as the periaqueductal grey matter (PAG) and the dorsolateral pons (DLP), in migraine attacks: the ‘migraine generator’. In addition, a series of laboratory experiments have proposed that the brainstem might act as a driver of changes in cortical activity during migraine [21, 22]. Although the validity of the brainstem generator theory has been widely debated in the last few years [23], the role of relevant brainstem nuclei—including the rostral ventral medulla, the locus coeruleus, the superior salivatory and cuneiform nucleus—in modulating trigeminovascular pain transmission and autonomic responses in migraine is well established [4, 18, 19]. Furthermore, there is evidence showing antimigraine drugs such as triptans [24, 25], ergot derivatives [26, 27] and the novel CGRP receptor antagonists [28, 29] can specifically modulate activity in the TCC, which might explain their effect in aborting migraine.

### The hypothalamus

The central role of the hypothalamus in cluster headache and other trigeminal autonomic cephalalgias is well established [30–32]. Several studies have recently highlighted its possible involvement in migraine as well. Evidence shows that this brain structure has direct and indirect anatomical connections to the thalamus [33], trigeminovascular neurons [34, 35] and sympathetic and parasympathetic brainstem nuclei [36], supporting its role in nociceptive and autonomic modulation in migraine patients. Previous positron emission tomography studies have shown hypothalamic activation during spontaneous migraine headache [37] and during the premonitory phase [38]. Recently, Schulte and May performed an elegant study in which a migraine patient underwent functional neuroimaging for 30 consecutive days. During the 24 h preceding the attack as well as throughout the ictal phase an altered functional connectivity between the hypothalamus and the areas of the brainstem generator was found, leading the authors to hypothesize that this network change might be the real driver of attacks [39]. The key involvement of the hypothalamus in migraine explains symptoms that begin in the early ictal stages and last throughout the attack, such as craving, mood changes, yawning and fatigue [4, 40].

### The thalamus

The thalamus is a nociceptive relay station where inputs from the dura mater as well as cutaneous regions are conveyed through second-order trigeminovascular neurons. It is a central area for the processing and integration of pain stimuli and its connection to a wide variety of cortical areas such as the somatosensory, motor, visual, auditory, olfactory and limbic regions can explain part of the complexity of migraine features [41]. Thalamo-cortical transmission is constantly modulated by different pathways involved in cognition, emotion and autonomic responses [42]. Several studies have reported structural [43–45] and functional [19, 46–49] thalamic alterations in migraineurs during the ictal and interictal phase, which can be detected since the paediatric age and might influence the onset of the migraine attack. Furthermore, the thalamus has shown to be a pivotal area for the development of sensory hypersensitivity to visual stimuli [50] and mechanical allodynia [51].

Several acute [24] and preventive [52–55] migraine therapies are thought to act centrally through the modulation of thalamic neurons. Recently, Andreou et al. [56] showed that the efficacy of single pulse transcranial magnetic stimulation (sTMS) in the treatment of migraine with and without aura [57] might be related not only to its capability to block cortical spreading depression (CSD) but also to its modulation of thalamo-cortical activity.

### The cortex

Even if the role of the cortical wave of spreading depression first identified by Leão [58, 59] in the generation of aura is well established [60, 61], its activity as a potential trigger for migraine headache is less clear. Those in favour of this theory argue that experimental studies in rats have shown that CSD can trigger neurogenic meningeal inflammation and subsequently activate the trigeminovascular system [62, 63]; however, this has not been confirmed in humans. Many changes in the structure and function of key cortical areas have been reported over the last years in migraine patients both with and without aura. Specifically, cortical changes in the ictal and interictal period have been shown in regions normally associated with pain processing such as the insular, somatosensory, prefrontal, and cingulate cortex [64, 65].

A large body of evidence has pointed to an increased sensitivity to different sensory stimuli in migraineurs during the attack and in the interictal phase [66]. In addition, several neurophysiological studies have reported a reduction of the common physiological response known as habituation, in which repeated stimulations cause a decrement in the amplitudes of sensory responses [67, 68]. The lack of habituation in migraine, measured for different sensory modalities, usually occurs during the pain-free period and reverts during the ictal phase or with attacks becoming more frequent [66]. Although the neural mechanisms underlying sensitization and habituation deficits remain poorly understood, the presence of a widespread cortical dyshabituation has been hypothesized as one of the main contributors to this deficit [69].

Recent large genome-wide association studies have identified 13 susceptibility gene variants in migraine patients which are mainly involved in glutamatergic neurotransmission and synaptic plasticity, and whose impairment may, therefore, be considered a key mechanism underlying an abnormal cortical excitability [70, 71].

Finally, positive results from the use of novel therapeutic approaches capable of modulating neuronal activity in the cortex also confirm the possibility of an abnormal cortical responsivity in migraine [56], as will be highlighted further.

## Novel therapies in migraine

Migraine therapy has historically been divided between acute and preventive treatments, a structure that for simplicity is followed in this review. It is, however, becoming evident that this dichotomous principle might in fact be dated [2], especially by observing the mechanism of action of novel migraine therapies such as the CGRP antagonists, which have been studied as both acute and preventive migraine agents.

### Acute therapies

Treatment for the acute migraine attack ranges from nonspecific medications—such as non-steroidal anti-inflammatory drugs and combination analgesics—to migraine-specific drugs, including ergotamine preparations and triptans. Triptans, which act by targeting 5-HT_1B_ and 5-HT_1D_ serotonin receptors, were the first drugs specifically developed as acute migraine therapies [72]. Although they can be very effective in many individuals, they often have significant limitations to their use caused by adverse effects. Furthermore, lack of efficacy and recurrence of migraine symptoms are seen in over 50% of cases in most studies [73, 74]. As a consequence, in the last years there has been a search for promising novel therapeutic agents to better treat migraine patients.

CGRP is a neuropeptide widely expressed in both peripheral and central neurons. Aside from its action as a potent cerebral arteriolar dilatator, substantial evidence has pointed to its role in modulating central and peripheral pain circuits. Studies showing the mediating action of CGRP on second- and third-order neurons seem to underline its regulatory role in central pain mechanisms. Furthermore, elevation of this molecule in migraineurs is thought to be linked to a decrease in descending inhibitory mechanisms which in turn might lead to migraine susceptibility through sensitization of multiple central neuronal circuits [8]. These findings have progressively led to the development of new drugs that target the CGRP pathway. Six different CGRP receptor antagonists, the gepants, have been developed for use in acute migraine [72]. Remarkably, each study reported positive outcomes on the primary endpoint of pain freedom when comparing the new drugs to placebo. However, two studies were stopped due to liver toxicity [75, 76] and three because of lack of interest from the companies [77–79]. One study testing the molecule ubrogepant is currently in phase III [80]. Notably, these medicines have a better tolerability in terms of central nervous system and vascular side effects compared to triptans and they seem to present a lower risk of causing medication overuse [2, 73].

Another encouraging new acute treatment for migraine is represented by the drug class of 5-HT_1F_ receptor agonist called ditans. Several studies have shown that 5-HT_1F_ receptors are not expressed in the vasculature [81] and that ditans inhibit activation of cells in the trigeminal nucleus caudalis evoked by trigeminal stimulation [82, 83]. Lasmiditan has been studied in two randomized, placebo-controlled double-blind trials which showed significant improvement, measured in terms of headache freedom at 2 h [84, 85], with its use. The main advantage of this new drug is the lack of any cardiovascular and cerebrovascular effects [86], although mild side effects such as dizziness, fatigue, vertigo and somnolence have been reported in the randomized controlled trials (RCT).

Glutamatergic targets, including both metabotropic and ionotropic glutamate receptors, are also expected to have a prominent role in future migraine therapy. Recent experimental and clinical studies have shown an effect of NMDA, AMPA, iGluR5 and mGluR5 receptor antagonists in migraine, although their efficacy was lower than that of sumatriptan and related visual side effects were observed [87–89]. The NMDA receptor, however, could prove to be an important target for the management of migraine with aura, as shown by small RCT testing the effects of ketamine in reducing the severity of auras [90].

### Preventive therapies

Preventive therapies are recommended in patients with chronic migraine and in more than a third of episodic migraine patients, especially in the case of frequent attacks or in subjects who do not tolerate and respond to acute treatments [91]. Many drugs of different pharmacological categories—such as β blockers, anticonvulsants, tricyclic antidepressants and calcium channel modulators—have been approved for migraine prevention or have class A evidence supporting their use. Patients’ compliance and adherence to these medications, however, is often poor due to their modest efficacy and adverse effects [86]. Therefore, more effective and better tolerated drugs are currently being studied for preventive use in migraine, mainly represented by monoclonal antibodies (mAB) to either CGRP peptide (galcanezumab, eptinezumab or TEV-48125) or its canonical receptor (erenumab). Data from a total of five RCTs performed on episodic migraine patients [92–96] revealed that these compounds present a therapeutic gain—measured through 50% responder rates for migraine/probable migraine days—ranging from 17 to 31. In the two placebo-controlled RCTs for chronic migraine [97, 98] the therapeutic gain was of 16 and 24 [2]. Even though monoclonal antibodies are very likely to represent the future strategy for effective migraine prevention, there are several caveats to their use that need to be considered. First, given the relatively short duration of the ongoing studies, evidence is needed to exclude long-term issued linked to the use of mAB. Furthermore, there is little knowledge regarding the development of autoantibodies against these compounds following prolonged treatment. Lastly, the elevated cost of these molecules must be counterbalanced by a high patient benefit to justify their extensive use.

Other targets for migraine therapy focusing on the supposed pathophysiological role of neuroinflammation in inducing migraine attacks—such as substance P, neurokinin 1 receptors [99] and orexin receptors [100]—have consistently failed in clinical trials in recent years. This evidence once more suggests that targeted migraine therapies must focus on specific neuronal mechanisms [2, 86].

### Neuromodulation

Neuromodulation is a promising approach that has emerged in recent years with both acute and preventive migraine treatment strategies. These exciting techniques range from invasive approaches such as occipital nerve stimulation (ONS) and sphenopalatine ganglion (SPG) stimulation, which have been used for several years and are largely positioned in intractable chronic patients, to more modern non-invasive devices that target the nervous system transcutaneously. The latter are mainly represented by TMS, non-invasive vagus nerve stimulation (nVNS), supraorbital nerve stimulation and transcranial direct current stimulation (tDCS).

ONS has been investigated as a prevention in chronic migraine patients in three randomized controlled trials: each was negative [101–103]. A later open-label follow-up study has shown a modest 12-month efficacy rate of ONS for headache pain and disability, although the complication rates associated with this procedure were still high [104].

Several experimental studies have demonstrated that the SPG has connections with the trigeminovascular system [105], explaining the presence of cranial autonomic symptoms in primary headaches and suggesting a potential role for the SPG in pain modulation [106]. Preliminary studies reported an improvement in pain intensity after lidocaine-induced SPG block [107, 108] or electrical SPG stimulation [109] during acute migraine attacks. In addition, a trend of reduction in migraine days per month and an amelioration in several quality of life measures were reported after repetitive SPG blockades with 0.5% bupivacaine [110]. Two RCTs are currently evaluating the acute use of a surgically implanted SPG neurostimulator in high disability migraine (NCT01540799, NCT01294046) and results are awaited. The positive results of a double-blind, randomized, sham-controlled trial performed on 67 episodic migraine patients (the PREMICE study) [111] followed by an audit on more than 2000 patients [112] have led to the approval of the non-invasive transcutaneous supraorbital nerve stimulator (Cefaly^®^) as a preventive treatment for migraine. A current RCT (NCT02590939) is testing the Cefaly^®^ device as an acute treatment; however, further studies with a focus on blinding issues are needed to confirm its efficacy as a preventive treatment in migraine.

Early studies on patients with comorbid epilepsy or depression and headache supported a possible effect of vagus nerve stimulation in migraine. Different open-label studies for the treatment of acute migraine attacks using a novel portable device for nVNS (GammaCore^®^) demonstrated that its effect was comparable to that of most commonly used triptans with mild and well-tolerated side effects [113–115]. Regarding its preventive use, a double-blind, sham-controlled study in chronic migraine patients revealed a modest reduction in headache days in the active group compared to the sham group after two months (−1.4 vs. −0.2 days; *p* = 0.56) [116]. However, the open-label extension data suggests that longer term use of nVNS might be effective. Another recent open-label study on menstrual-related migraine reported a significant reduction in the number of migraine days and analgesic use following a 12-week treatment period in 56 patients [117].

On the basis of previous experimental studies [118] and recent evidence [56] supporting a positive effect of sTMS in inhibiting CSD and the activity of thalamo-cortical neurons, a handheld device (SpringTMS^®^) has been recently developed and approved for the treatment of acute migraine attacks. A preliminary multicentre, randomized, double-blind, parallel-group, sham-controlled study [57] on 164 migraine patients with aura demonstrated a superiority of sTMS over sham stimulation for pain freedom at 2 h (39 vs 22%, *p* = 0.018) and for sustained pain freedom at 24 (29 vs 16%, *p* = 0·04) and 48 (27 vs 13%, *p* = 0·03) hours. Moreover, a post-marketing phone-based survey [119] on 190 episodic and chronic migraine patients revealed that 62% had a reduction in their migraine headaches and 59% reported a decrease in the number of headache days after a 12-week treatment. There is, however, still a lack of large controlled RCTs to support the use of the sTMS for the prevention of migraine.

Another neuromodulation approach has focused on the application of repeated cathodal or anodal transcranial direct current stimulation over the visual cortex, although data on its therapeutic effect in migraineurs have been conflicting [120, 121].

It is clear from the available evidence that although very promising, neuromodulation techniques require further studies to confirm their efficacy in migraine.

## Conclusions

The recent recognition of migraine as a debilitating neurological condition is an important advance in directing more resources to the development of new treatments and their deployment to patients. The last two decades have seen a number of important studies in the area of primary headaches leading to an extremely exciting era for researchers interested in this disorder. New treatments are rapidly becoming available for patients and a better understanding of its pathophysiological mechanisms is allowing a greater awareness of the complexity of a brain disease which has often been overlooked and under-managed.
